# Quantification of anticholinergic and sedative drug load with the Drug Burden Index: a review of outcomes and methodological quality of studies

**DOI:** 10.1007/s00228-016-2162-6

**Published:** 2016-12-01

**Authors:** Hans Wouters, Helene van der Meer, Katja Taxis

**Affiliations:** Department of PharmacoTherapy, -Epidemiology & -Economics [PTEE], Groningen Research Institute of Pharmacy, Faculty of Mathematics and Natural Sciences, University of Groningen, Antonius Deusinglaan 1, 9713 AV Groningen, Netherlands

**Keywords:** Antimuscarinic agents, Hypnotics and sedatives, Polypharmacy, Older adults, frailty, Inappropriate prescribing

## Abstract

**Purpose:**

The Drug Burden Index (DBI) is a non-invasive method to quantify patients’ anticholinergic and sedative drug burden from their prescriptions. This systematic review aimed to summarise the evidence on the associations between the DBI and clinical outcomes and methodological quality of studies.

**Methods:**

A search in PubMed and Embase (search terms: ‘drug’, ‘burden’, and ‘index’) was performed and experts were contacted. We excluded publications that did not report empirical results or clinical outcomes. Methodological quality was assessed using the Newcastle-Ottawa Scale. Potential omissions of relevant clinical outcomes and populations were studied.

**Results:**

Of the 2998 identified publications, 21 were eligible. Overall, methodological quality of studies was good. In all but one study, adjustment was made for prevalent co-morbidity. The DBI was examined in diverse older individuals, i.e. both males and females from different settings and countries. However, no studies were conducted in other relevant patient groups, e.g. psychiatric patients. Exposure to anticholinergic and sedative drugs was thoroughly ascertained, though the specific calculation of the DBI differed across studies. Outcomes were assessed from medical records, record linkage or validated objective tests or questionnaires. Many studies found associations between the DBI and outcomes including hospitalisation, physical and cognitive function. Cognitive function and quality of life were understudied and the number and scope of longitudinal studies was limited.

**Conclusions:**

An accumulating body of evidence supports the validity of the DBI. Longitudinal studies of cognitive function and quality of life and in other patient groups, e.g. psychiatric patients, are warranted.

**Electronic supplementary material:**

The online version of this article (doi:10.1007/s00228-016-2162-6) contains supplementary material, which is available to authorized users.

## Introduction

Drugs with anticholinergic and sedative properties are prescribed in over a quarter of older patients [1, 2] despite their likelihood to increase patients’ physical and cognitive impairment [3–5]. In 2007, Hilmer and colleagues published the Drug Burden Index (DBI) [6], a linear additive model that quantifies cumulative anticholinergic and sedative drug load for patients with polypharmacy:

$$ DBI=\sum \frac{D}{\delta +D} $$

where *D* is the daily dose of an individual drug and *δ* usually represents the minimum recommended daily dose of that individual drug. The sigma sign (*∑*) indicates that the DBI is the sum score of prescribed drugs with probable anticholinergic and sedative properties for each patient. Compared to other methods of estimating patients’ anticholinergic and sedative drug load such as serum anticholinergic activity (SAA) [7] and related scales such as the Anticholinergic Risk Scale (ARS) [8] and the Anticholinergic Drug Scale (ADS) [9], the DBI has two advantages. First, it is non-invasive as it is calculated from drug prescriptions. Unlike SAA, the DBI does not require blood withdrawal. Second, the DBI takes the dosage of each anticholinergic and sedative drug into account whereas the ARS and ADS scales do not. Thus, these two advantages clearly favour the DBI over other measures as a tool for the routine screening of anticholinergic and sedative burden in the process of deprescribing of anticholinergic and sedative drugs by pharmacists and physicians [10–12].

A growing body of studies conducted in Australia, North America, and Europe have examined associations between the DBI and clinical outcomes. Recently, Kouladjian et al. [13] discussed these findings in a comprehensive overview of studies, thereby providing insight into the clinical and theoretical applications of the DBI. There is increasing interest in methodological issues about how to estimate exposure to anticholinergic and sedative drugs. [14–17] Two recent systematic reviews evaluated the scales that are currently used to quantify the anticholinergic and sedative drug burden as well as associated clinical outcomes. [18, 19] These systematic reviews found drugs with anticholinergic properties to increase the risks of cognitive impairment, falls, functional outcomes and all-cause mortality in older adults.

The current systematic review aims to assess the scope and quality of studies about the DBI focusing on methodology, patient populations and outcomes. In doing so, the current systematic review aims to complement these previous systematic reviews. Such an assessment is needed to reflect on current knowledge about the associations between patients’ DBI values and clinical outcomes, to provide advice on methodological requirements for future studies and to identify knowledge gaps about clinically relevant outcomes.

## Material and methods

### Data sources and search strategy

We conducted a search and extraction according to the PRISMA statement [20] in September 2015. We searched for all English publications about original studies of the DBI since its launch in 2007 with the search term: Drug [All Fields] AND Burden [All Fields] AND Index [All Fields] AND [“2007/01/01”[PDAT] : “2015/09/30”[PDAT]]) in PubMed and Embase and in the reference lists of initially found publications. Thus, the search was not limited to studies conducted in older adults. Authors whose publications were not electronically available were contacted and requested to provide their publications. Lastly, we contacted experts from relevant publications to identify other studies.

### Study screening and selection

Publications were included if they reported empirical results and associations between the DBI and clinical outcomes. Publications were excluded if they were not available either electronically or from the authors. Publications were not excluded if the DBI calculation was based on only anticholinergic or sedative drugs.

### Data extraction and synthesis

Eligibility, methodological quality and study outcomes were extracted by HW and reviewed by HVDM. In case of disagreements between HW and HVDM, final decisions were made by KT. For studies in which separate DBIs were calculated for anticholinergic and sedative drugs, results of both DBIs were considered. If studies reported both a standard overall DBI calculated for anticholinergic and sedative drugs and DBIs calculated separately for anticholinergic and sedative drugs, only results of the standard overall DBI were considered.

All eligible studies were rated for their methodological quality using the Newcastle-Ottawa Scale [NOS] [21]. The NOS awards stars with regard to *selection* of participants, i.e. representativeness (*1 star*), and selection of participants not exposed to anticholinergic and sedative drugs (controls) (*1 star*), *ascertainment* of anticholinergic and sedative exposure (*1 star*), *comparability* of participants with high and low DBI values, i.e. by taking the most important confounding factor (*1 star*) and additional confounding factors (*1 star*) into account, and *outcomes*, i.e. whether these were assessed in a blind manner (*1 star*). Furthermore, for longitudinal studies, whether incidence of outcomes was assessed or whether their absence at baseline was verified or adjusted for (*1 star*), and *adequacy of follow-up*, i.e. whether length of follow-up was adequate *(1 star)*, and whether the amount of attrition, and presence of differential attrition or selective loss to follow-up, e.g. more loss to follow-up among patients with higher baseline frailty, was assessed and acceptable *(1 star)*. Furthermore, we also assessed how often relevant clinical outcomes were studied and potential omissions of relevant clinical outcomes, as well as the specific patient populations that were studied.

## Results

**Fig. 1:**
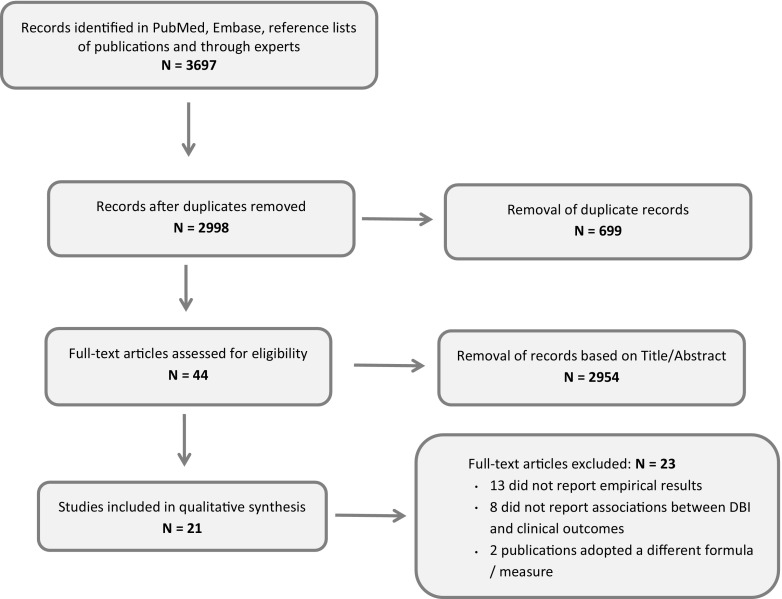
Flowchart of identification and assessment of eligibility of DBI publications

The search resulted in 2998 publications. After exclusion of 2954 publications based on title and abstract, and excluding 23 publications after reviewing their full texts, 21 eligible publications were included in the review (see flowchart in Fig. 1). Experts confirmed there were no other eligible publications. Owing to the substantial heterogeneity with regard to clinical outcomes, classification of DBI values, and study design, we decided that a qualitative synthesis of the literature was more suitable than a quantitative meta-analysis. Key characteristics of eligible publications are presented in Table 1.

**Table 1:** Characteristics of eligible publications of cross-sectional and longitudinal studies (*N* = 21)

Publication *1st author et al. year*)	Sample size	Data source and country	Setting	Participants’ characteristics		
				% women	Age	Number of medicines
Cross-sectional studies						
Best et al. 2013 [22]	329	I, Australia	Hospital wards	62	85 [**±**7]	8 [**±**4]
Bosboom et al. 2012 [23]	226	II, Australia	RACF	75	86 [**±**8]	10 [**±**4]
Cao et al. 2008 [24]	932	III, USA	Community	100	78 [71–86]^a^	–
Gnjidic et al. 2012a [25]	700	IV, Finland	Community	69	81 [**±**5]	5 [**±**3]
Gnjidic et al. 2009 [26]	1705	V, Australia	Community	0	77 [**±**6]	4 [**±**3]
Gnjidic et al. 2012b [27]	115	VI, Australia	Community	73	82 [**±**6]	5 [**±**3]
Gnjidic et al. 2012c [28]	987	V, Australia	Community	0	77 [**±**6]	4 [**±**3]
Hilmer et al. 2007 [6]	3075	VIII, USA	Community	52	74 [**±**3]	3 [**±**3]
Lowry et al. 2012 [29]	362	X, UK	Geriatric wards	59	84 [**±**7]	7 [5–9]^a^
Mangoni et al. 2013 [30]	71	XI, Holland	Hospital wards	70	84 [**±**6]	4 [**±**3]
Longitudinal studies						
Dauphinot et al. 2014 [31]	337	XIV, France	Geriatric wards	65	85 [**±**7]	7 [**±**4]
Gnjidic et al. 2012d [32]	1662	V, Australia	Community	0	77 [**±**5]	4 [±3]
Gnjidic et al. 2014 [33]	33,206	VII, Finland	Community	67	79 [**±**9]	–
Hilmer et al. 2009 [34]	2172	VIII, USA	Community	53	73 [**±**3]	–
Kashyap et al. 2014 [35]	102	IX, Canada	Community	84	72 [**±**7]	7 [**±**4]
Lönnroos et al. 2012 [36]	339	IV, Finland	Community	68	81 [**±**5]	5 [**±**3]
Nishtala et al. 2014 [37]	537,387	XII, New Zealand	Community	55	75 [**±**7 ]	6 [**±**4]
Salahudeen et al. 2015 [38]	537,387	XII, New Zealand	Community	55	75 [**±**8]	6 [**±**4]
Wilson et al. 2010 [39]	602	XIII, Australia	RACF	71	86 [**±**6]	6 [**±**3]
Wilson et al. 2011 [40]	602	XIII, Australia	RACF	71	86 [**±**6]	6 [**±**3]
Wilson et al. 2012 [41]	602	XIII, Australia	RACF	71	86 [**±**6]	6 [**±**3]

Data sources: I, Concord Repatriation General Hospital Sydney New South Wales Australia; II, DIRECT Study Beer C et al. Trials 2010; 11: 63; III, Medicare beneficiaries Baltimore City and Baltimore Country, Maryland; IV, GeMS study Rikala et al., Drugs Aging. 2010; 27:337–49; V, CHAMP, Cumming et al., Int J Epidemiol 2008; 38: 374–8; VI, Self-care retirement villages in Sydney, Australia; VII, Linkage of Finnish National Prescription and Special Reimbursement Registers with Finnish Hospital Discharge Register; VIII, Health ABC study community-resident Medicare recipients, Pittsburgh, Pennsylvania and Memphis, Tennessee; IX, Patients of incontinence clinics, Montreal and Sherbrooke areas of Quebec, Canada; X, Two acute geriatric medicine units from Aberdeen Royal Infirmary and Woodend Hospital, NHS Grampian, Aberdeen, Scotland, United Kingdom; XI, Academic Medical Centre, Amsterdam, the Netherlands; XII, Pharmaceutical Claims Data Mart [Pharms] of the Pharmaceutical Management Agency [PHARMAC] and data from the Ministry of Health of New Zealand; XIII, Multicentre cluster-randomised controlled trial of RACFs residents in the Northern Sydney Central Coast Health [NSCCH] service area, Australia; XIV Consecutive sample of hospitalised patients of three geriatric hospitals, University Hospital of Lyon, France

*RACF* residential aged care facility

^a^Median value with interquartile range

### Selection of participants and populations studied

Ratings of the methodological quality of eligible studies are presented in Online Resource 1. All studies focused on older geriatric patients as the mean age of study populations ranged from 72 to 86 years. Furthermore, sampling in several studies was found to be restricted to a single urban area [25, 27, 31, 36], to participants from RCTs [23, 39–41], to either female [24] or male participants [26, 28, 32] or to a single or two recruitment sites [22, 29, 30]. Other studies were more representative of geriatric patients [6, 34, 35], very large [33], or even studied a national sample of older individuals [37, 38]. Overall representativeness of studies was good as individual studies included both male and female participants were conducted in different settings, i.e. hospital wards, residential aged care facilities or community-dwelling older people. Studies were also conducted in various countries although these were predominantly countries from Australasia, Europe and North America (Table 1). Individuals who were not exposed to anticholinergic and sedative drug load were consistently drawn from the same population as exposed individuals.

### Ascertainment of anticholinergic and sedative exposure

Exposure to drugs was thoroughly ascertained in the majority of studies, thus providing a substantial base for the DBI calculation. Drug exposure was assessed through participants’ self-report during a structured interview and a verification of participants’ answers through inspection of prescription forms and packages by qualified assessors [6, 24–28, 32, 34–36], through dispensing data on medication [33, 37, 38] or from clinical records [22, 23, 29–31, 39–41]. In some studies, the DBI was calculated for anticholinergic and/or sedative drugs separately [24, 30, 35].

However, some caveats were also observed. Although the grounds were mentioned for classifying drugs as being anticholinergic or sedative, none of the lists of DBI drugs has been published. Furthermore, studies conducted in the USA used the minimum recommended daily dose as approved by the US Food and Drug Administration [6] while studies outside the USA used other national reference sources to estimate the *δ* or the minimum daily dose. Possible differences between studies and the influence of such differences on associations between patients’ DBI values and clinical outcomes could not be assessed. One study examined the relationship between the DBI and SAA but found no significant relationship [30]. Other studies compared the DBI with other anticholinergic scales [35] or the Beers criteria [27].

### Comparability of participants with high and low DBI values

In all but one study, [35] adjustment was made for prevalent co-morbidity. In all studies, age was adjusted for in relevant analyses and studies that included men and women also adjusted for sex. Several of these studies also adjusted for cognitive impairment, or presence of dementia [22, 23, 25–27, 29, 30, 36, 39, 40] and depressive or other neuropsychiatric symptoms including sleep problems [23, 24, 26–28, 34, 39, 40]. If cognitive function was the outcome, analyses had most of the time been adjusted for age [6, 24, 28, 39] and educational level [6, 24, 28], which are important determinants of cognitive function. Five studies adjusted for prescribed drugs other than those included in the DBI calculation [22, 24, 29, 40, 41]. In one study, patients and controls were matched for age, sex and region of residence [33].

### Outcomes

Outcomes were usually assessed through record linkage, e.g. national prescription or reimbursement registers and hospital discharge registers [30, 33, 36–38], medical records and clinical notes [22, 31, 40, 41] or through objective tests (see below).

Tables 2 and 3 present the associations found in different studies between the DBI and various clinical outcomes. Across different studies, the DBI was either studied as a continuous or categorised measure.

**Table 2:** Associations between the Drug Burden Index [DBI] and mortality, healthcare utilisation and falls

Outcome category	S/NS	Outcome	DBI categorisation	Statistic
Mortality				
Dauphinot et al., 2014 [31]	NS	In-hospital mortality	DBI DDD increase	HR, 1.9 [95% CI: 0.8–4.4]^a^
Gnjidic et al., 2014 [33]	S	Mortality AD patients	Continuous	HR: 1.21 [95% CI: 1.09–1.33]
	S	Mortality non-AD patients	Continuous	HR: 1.37 [95% CI: 1.20–1.56]
Nishtala et al., 2014 [37]	S	Mortality	DBI [>0]	HR: 1.29 [95% CI: 1.25–1.33]
Wilson et al., 2012 [41]	NS	Mortality	DBI [0–1]	HR: 1.13 [95% CI: 0.82–1.57]
	NS	Mortality	DBI [≥1]	HR: 1.19 [95% CI: 0.82–1.74]
Mangoni et al., 2013 [30]	S	1-year mortality	Anticholinergics	HR: 3.2 [95% CI: 1.1–9.4]^a^
Hospitalisation and GP visits				
Best et al., 2013 [22]	NS	Delirium related	DBI 0–1	OR: 1.43 [95% CI: 0.79–2.62]
	S	Delirium related	DBI [≥1]	OR: 2.95 [95% CI: 1.34–6.51]
	NS	Fall related	DBI 0–1	OR: 1.30 [95% CI: 0.74–2.28]
	NS	Fall related	DBI [≥1]	OR: 1.52 [95% CI: 0.70–3.30]
	NS	Length of stay	DBI 0–1	OR: 0.98 [95% CI: 0.59–1.63]
	NS	Length of stay	DBI [≥1]	OR: 0.74 [95% CI: 0.37–1.49]
Gnjidic et al., 2014 [33]	NS	Length of stay AD patients	Continuous	IRR: 1.06 [95% CI: 0.99–1.12]
	S	Length of stay non-AD patients	Continuous	IRR: 1.35 [95% CI: 1.24–1.46]
	S	No. admissions AD patients	Continuous	IRR: 1.22 [95% CI: 1.17–1.27]
	S	No. admissions non-AD patients	Continuous	IRR: 1.36 [95% CI: 1.28–1.43]
Lowry et al., 2012 [29]	S	Length of stay	Continuous	HR: 1.23 [95% CI: 1.06–1.42]
Lönnroos et al., 2012 [36]	NS	Days per person-year	DBI 0–1	RR: 1.10 [95% CI: 0.53–2.28]
	NS	Days per person-year	DBI ≥ 1	RR: 0.80 [95% CI: 0.29–2.22]
Nishtala et al., 2014 [37]	S	Fall-related hospitalisation	DBI > 0	IRR: 1.56 [95% CI: 1.48–1.65]
	S	GP visits	DBI > 0	IRR: 1.13 [95% CI: 1.12–1.13]
Salahudeen et al., 2015 [38]	S	Hospital admission	Continuous	IRR: 1.36 [95% CI: 1.31–1.42]
	S	Fall related	Continuous	IRR: 1.59 [95% CI: 1.46–1.74]
	S	Length of stay	Continuous	IRR: 1.50 [95% CI:1.44–1.56]
	S	GP visits	Continuous	IRR: 1.26 [95% CI:1.25–1.27]
Falls				
Dauphinot et al., 2014 [31]	S	During hospital stay	DBI WHO increase	HR: 2.85 [95% CI: 1.14–7.12]
Wilson et al., 2011 [40]	S	12-month study period	DBI 0–1	IRR: 1.61 [95% CI: 1.17–2.23]
	S	12-month study period	DBI ≥ 1	IRR: 1.90 [95% CI: 1.30–2.78]

*S* significant, *NS* not significant, *HR* hazard ratio, *OR* odds ratio, *IRR* incidence rate ratio, *RR* relative risk, *AD* Alzheimer’s disease, *DDD* defined daily dose, *WHO* World Health Organisation

^a^Not adjusted for covariates

**Table 3:** Associations between the Drug Burden Index [DBI] and physical and cognitive function, and quality of life

Outcome category	S/NS	Outcome	DBI categorisation	Statistic
Physical function and IADL				
Cao et al., 2008 [24]	S	Mobility difficulty	Anticholinergics	OR: 3.2 [95% CI: 1.5–6.9]
	S	Slow gait	Anticholinergics	OR: 3.6 [95% CI: 1.6–8.0]
	S	Balance difficulty	Anticholinergics	OR: 4.9 [95% CI: 2.0–12.0]
	S	Chair stands	Anticholinergics	OR: 4.2 [95% CI: 2.0–8.7]
	S	Grip strength	Anticholinergics	OR: 2.4 [95% CI: 1.1–5.3]
	S	Upper extremity	Anticholinergics	OR: 2.7 [95% CI: 1.3–5.4]
	S	ADL	Anticholinergics	OR: 3.4 [95% CI: 1.7–6.9]
	S	Mobility difficulty	Sedatives	OR: 2.4 [95% CI: 1.1–5.3]
	NS	Slow gait	Sedatives	OR: 0.9 [95% CI: 0.4–1.9]
	NS	Balance difficulty	Sedatives	OR: 1.7 [95% CI: 0.7–4.0]
	NS	Chair stands	Sedatives	OR: 1.8 [95% CI: 0.8–3.9]
	S	Grip strength	Sedatives	OR: 3.3 [95% CI: 1.5–7.3]
	NS	Upper extremity	Sedatives	OR: 2.0 [95% CI: 1.0–4.2]
	NS	ADL	Sedatives	OR: 1.2 [95% CI: 0.6–2.2]
Gnjidic et al., 2012a [25]	S	10-m walking speed	DBI > 0	B: −0.13 [95% CI: −0.19, −0.08]
	S	Chair stands	DBI > 0	B: 1.11 [95% CI: 1.05, 1.16]
	S	TUG	DBI > 0	B: 1.13 [95% CI: 1.07, 1.19]
	S	IADL	DBI > 0	B: −0.61 [95% CI: −0.84, −0.39]
	S	ADL	DBI > 0	B: −3.21 [95% CI: −4.68, −1.75]
	NS	Grip strength	DBI > 0	B: −0.98 [95% CI: −2.05, 0.08]
Gnjidic et al., 2009 [26]	NS	Chair stands	DBI > 0	B: 0.58 [95% CI: −0.11, 1.27]
	S	Walking speed	DBI > 0	B: −0.03 [95% CI: −0.05, −0.00]
	S	Narrow walk speed	DBI > 0	B: −0.03 [95% CI: −0.05, −0.01]
	S	Balance difficulty	DBI > 0	B: −0.11 [95% CI: −0.18, −0.03]
	S	Grip strength	DBI > 0	B: −1.09 [95% CI: −1.90, −0.28]
	S	IADL	DBI > 0	B: 0.18 [95% CI: 0.04, 0.32]
Gnjidic et al., 2012b [27]	S	SPPB	Continuous	B: −1.28 [95% CI: −2.53, −0.04]
	NS	Grip strength [kg]	Continuous	B: 0.10 [95% CI: −2.54, 2.74]
Hilmer et al., 2009 [34]	S	SPPB	AUCDB	B: −0.08, *t* value: 2.46, *p* < .01
	S	Gait speed	AUCDB	B: −0.01, *t* value: −2.86, *p* = 0.004
	S	Grip strength	AUCDB	B: −0.27, *t* value: −2.87, *p* = .004
Hilmer et al., 2007 [6]	S	Health ABC performance score	Continuous	B: −0.15, *t* value: −5.73, *p* < .001
Lowry et al., 2012 [29]	S	Barthel Index	Continuous	OR: 0.71 [95% CI: 0.55–0.91]
Wilson et al., 2010 [39]	S	Balance	AUCDB sedatives	OR: 1.57 [95% CI: 1.08–2.27]
Gnjidic et al., 2012d [32]	S	Prefrail	DBI > 0	OR: 1.62 [95% CI: 1.21, 2.15]
	S	Frail	DBI > 0	OR: 2.14 [95% CI: 1.25, 3.64]
Cognitive function				
Cao et al., 2008 [24]	S	MMSE	Anticholinergics	OR: 2.4 [95% CI: 1.1–5.1]
	NS	MMSE	Sedatives	OR: 1.1 [95% CI: 0.5–2.3]
Gnjidic et al., 2012c [28]	NS	ACE	DBI > 0	OR: 0.98 [95% CI: 0.66–1.47]
	NS	TMT	DBI > 0	OR: 0.71 [95% CI: 0.40–1.24]
	NS	Cognitive impairment	DBI > 0	OR: 1.34 [95% CI: 0.83–2.16]
Hilmer et al., 2007 [6]	S	DSST	Continuous	B: −1.51, *t* value: −2.50, *p* = .01
Kashyap et al., 2014 [35]	S	TMT-B	Anticholinergic	OR: 2.2 [95% CI: 1.1–8.06]^a^
	S	Delayed recall	Anticholinergic	OR: 4.2 [95% CI: 1.8–15.4]
Quality of life				
Bosboom et al., 2012 [23]	S	QoL	DBI > 0	B: −4.07 [95% CI: –7.25, −0.89]

*S* significant, *NS* not significant, *ACE* Addenbrooke’s Cognitive Examination, *ADL* activities of daily living, *DSST* digit symbol substitution test, *IADL* instrumental activities of daily living, *MMSE* Mini-Mental Status Examination, *QoL* quality of life, *SPPB* Short Physical Performance Battery, *TMT* Trailmaking Test, *TMT-B* Trailmaking Test part-B, *TUG* Time Up and Go test, *B* unstandardised regression coefficient, *OR* odds ratio

^a^Not adjusted for covariates

The majority of associations of the DBI with mortality, hospitalisation, falls, physical function and (instrumental) activities of daily living ([I]ADL), cognitive function and quality of life were statistically significant. Three of the five studies which assessed mortality and five of the six studies assessing hospital admissions found positive associations between the DBI and these outcomes. Higher DBI values were consistently found to be associated with increased fall risk. Impairments of physical function and IADL were examined in nine studies. Most studies consistently showed a higher DBI to be associated with several impairments with regard to mobility, balance difficulty, gait speed, IADL and ADL. Findings were equivocal for grip strength and chair stands. Compared to physical function, cognitive function was less frequently studied. Cognitive function was investigated in four studies using measures of global cognition and executive function, e.g. concentration and planning ability. Tests used were the Mini Mental Status Examination (MMSE) [24], specific tests [6, 39] or more extensive neuropsychological test batteries. [28, 35] Quality of life was also understudied as it was explicitly addressed in one study [23].

### Longitudinal studies

Eleven studies reported longitudinal results from eight patient cohorts. Incidence of outcomes was consistently taken into consideration, either through studying incidence of falls, frailty, GP visits, hospitalisation, mortality and physical function during a follow-up period [31–33, 36–38, 40, 41], through adjusting in the analyses for baseline physical function [34, 39] or through assessing change in cognitive function [35]. Studies with the shortest follow-ups, up to 12 months [36, 39, 40], had low attrition, being 4 and 13%, whereas those with a longer follow-up had attrition rates that not surprisingly ranged from 20% in a 2-year follow-up study [32] to ~30% in a 6-year follow-up study [34]. Although the ‘lost to follow-up’ rate of the latter study was substantial, it was associated with only minor differential attrition or selective loss to follow-up. An interesting modification of the DBI was the area under the curve for drug burden (AUCDB) or the average drug burden at each point in time multiplied by the time of exposure [34, 39]. This AUCDB enabled researchers to estimate the cumulative long-term exposure to anticholinergic and sedative drugs.

However, at the same time, the scope of the longitudinal studies was rather limited. They included registry data about ultimate outcomes such as mortality [31, 33, 37, 41], hospital admission, [33, 36–38] and falls [31, 40]. The number of prospective cohort studies that examined proximal outcomes, as directly assessed from patients themselves, was limited and addressed a limited number of outcomes of frailty, physical function, e.g. gait speed, grip strength, and balance and cognitive function [32, 34, 35, 39].

## Discussion

Overall, the studies of the DBI that have been conducted so far were of good methodological quality. Importantly, in all but one study, analyses were adjusted for co-morbidity. Although it is impossible to adjust for all confounding factors, adjusting for co-morbidity renders it unlikely that positive associations between the DBI and clinical outcomes simply reflected the treatment of multiple diseases or disorders with multiple drugs. The general picture of studies suggested that the DBI was examined in large to very large samples of older individuals who were diverse with respect to gender, residence and mean number of medicines prescribed. Exposure to drugs was in the majority of studies thoroughly ascertained through assessing medicine packages or dispensing data. Some longitudinal studies also adopted the AUCDB an adaptation of the DBI suitable for longitudinal research that takes the time of exposure to anticholinergic and sedative drugs into account. Differences between patients with high and low DBI values were adjusted for in analyses.

A large number of studies found associations between the DBI and relevant clinical outcomes. Impairments of physical function and IADL were most extensively examined. The physical measures provided interesting objective ‘proxy measures’ of fall risk particularly mobility and balance measures. Outcomes were assessed through medical records, validated tests or questionnaires. Longitudinal studies often had adequate follow-ups with attrition being either minor or not differential. Together, these findings support the use of the DBI in both research and clinical practice. In research, the DBI could serve as an important covariate that should be controlled for when examining, e.g. predictors of falling. In clinical practice, the DBI may be useful as a screener of frail patients to identify individuals with high anticholinergic and sedative exposure which might aggravate their physical and cognitive impairment. For example, a 1.5- to 3-fold increased risk of falling was observed for patients with high exposure to anticholinergic and sedative drugs compared to patients with no such exposure (see Table 2). Such patients are likely to be eligible for deprescribing interventions such as medication reviews conducted by pharmacists and general practitioners. In turn, screening with the DBI could be examined in controlled trials.

We have three suggestions about representativeness and selection of populations for further research. First, the DBI has been studied exclusively in older geriatric patient groups. However, the DBI could also be useful for other vulnerable but younger patient groups such as patients with psychiatric disorders and people with severe intellectual disabilities. Like in geriatric patients, these patients struggle with cognitive impairment. Polypharmacy with psychotropic medication is very common in people suffering from schizophrenia [42] and depression [43]. Patients often experience a high burden from the side effects of psychotropic medications [44]. Similar problems are known in people with severe intellectual disabilities [45, 46]. Second, studies of patients from other parts of the world including, e.g. China, the Middle East and South America are also worthwhile to pursue. Third, to further improve the clinical utility of the DBI for older individuals and in other vulnerable patient groups, more knowledge about the clinical implications of a certain DBI score is needed. In particular, whether this also depends on the underlying disease. DBI scores may carry a higher risk for patients who suffer from a degenerative disease such as Alzheimer’s disease, because of the loss of cholinergic neurons and the increased uptake of anticholinergic drugs in the brain due to increased permeability of the blood brain barrier [15].

A recommendation about the DBI calculation is that this should be based on a consensus list of medicines with anticholinergic and sedative properties which will be updated regularly, e.g. Duran et al. [47] have made attempts for a list of medications with anticholinergic properties. Also, the current DBI formula assumes that different drugs contribute linearly to the DBI score regardless of their potency. We suggest exploring the effects of weighing medication with high and low anticholinergic or sedative potency [15]. Finally, a potential source of bias is the high mortality rate in frail older people with high anticholinergic and sedative burden. This makes a careful examination of ‘differential attrition’ or selective loss to follow-up, owing to, e.g. baseline frailty, of even greater importance for cohort studies which enrol frail older people than for cohort studies in general.

Further research about the relationship between the DBI and cognitive function is needed. Specifically, for community-dwelling geriatric patients without dementia or early dementia as well as patients with psychiatric disorders, assessment of cognition with a standardised neuropsychological examination as was previously done [35] is needed in addition to an assessment of cognition with the MMSE [24] which has a diminished capacity to detect early cognitive impairment. [48] An extensive neuropsychological test battery allows a more sensitive assessment of a wide range of different cognitive functions. Furthermore, examination how the DBI relates to brain function using functional magnetic resonance imaging (fMRI) would be relevant in this regard. [49] For physical function tests, the opposite may actually be true, as Wilson et al. [39] argued that these tests might be too difficult for residents in RACFs who have advanced functional decline. For people with more advanced physical decline, selection of easier physical tests would be recommended. Moreover, quality of life could also be assessed [50] as a measure of general well-being.

This review had several strengths. The most important strength was our assessment of the methodological quality of studies using a standardised scale. Another strength was that the data extraction was reviewed by a second researcher. A possible limitation of our review was that it was not possible to conduct a quantitative meta-analysis, because of the limited number of studies, the wide array of clinical outcomes, the analysis of the DBI in different ways (i.e. continuous or dichotomous) and the use of country-specific minimum daily doses. Future meta-analyses, preferably individual patient data meta-analyses, should examine whether differences in study findings are associated with methodological differences between studies.

Thus, an accumulating body of evidence supports the validity of the DBI. What lies ahead are steps towards further refinement of the DBI in longitudinal studies aimed at substantiating the present body of evidence using an array of clinical outcomes in geriatric and other relevant patient groups.

### Authors’ contributions

Study design: HW KT

Data extraction: HW HVDM KT

Synthesis of findings: HW KT

Manuscript drafting: HW

Critical review of manuscript contents: HVDM KT

## Electronic supplementary material

ESM 1 (DOC 386 kb)
