# Supplementary material for: Quantification of anticholinergic and sedative drug load with the Drug Burden Index: a review of outcomes and methodological quality of studies
Source: Eur J Clin Pharmacol. 2016 Dec 1;73(3):257–66. doi: 10.1007/s00228-016-2162-6 (PMC5306241; doi:10.1007/s00228-016-2162-6)
Supplement: Supplementary file 1 — (DOC 386 kb) [file 228_2016_2162_MOESM1_ESM.doc]

| **Online Resource 1:** Methodological quality of eligible publications of cross-sectional and longitudinal studies * (N = 21) | | | | | | | | | | |
| --- | --- | --- | --- | --- | --- | --- | --- | --- | --- | --- |
| **Publication**  *(1st Author et al. & Year)* |  | **Selection^a^** Max. |  | **Ascertainment^b^**  Max. |  | **Comparability^c^**  Max. |  | **Outcomes^d^**  Max. |  |  |
|  |  |  |  |  |  |  |  |  |  |  |
| Cross Sectional Studies |  |  |  |  |  |  |  |  |  |  |
| Best et al. 2013 (1) |  |  |  |  |  |  |  |  |  |  |
| Bosboom et al. 2012 (2) |  |  |  |  |  |  |  |  |  |  |
| Cao et al. 2008 (3) |  |  |  |  |  |  |  |  |  |  |
| Gnjidic et al. 2012a (4) |  |  |  |  |  |  |  |  |  |  |
| Gnjidic et al. 2009 (5) |  |  |  |  |  |  |  |  |  |  |
| Gnjidic et al. 2012b (6) |  |  |  |  |  |  |  |  |  |  |
| Gnjidic et al. 2012c (7) |  |  |  |  |  |  |  |  |  |  |
| Hilmer et al. 2007 (8) |  |  |  |  |  |  |  |  |  |  |
| Lowry et al. 2012 (9) |  |  |  |  |  |  |  |  |  |  |
| Mangoni et al. 2012 (10) |  |  |  |  |  |  |  |  |  |  |
|  |  | **Selection^a^**  Max. |  | **Ascertainment^b^**  Max. |  | **Comparability^c^**  Max. |  | **Outcomes^d^**  Max. |  | **Follow-Up^e^**  Max. |
| Longitudinal Studies |  |  |  |  |  |  |  |  |  |  |
| Dauphinot et al. 2014 (11) |  |  |  |  |  |  |  |  |  |  |
| Gnjidic et al. 2012d (12) |  |  |  |  |  |  |  |  |  |  |
| Gnjidic et al. 2014 (13) |  |  |  |  |  |  |  |  |  |  |
| Hilmer et al. 2009 (14) |  |  |  |  |  |  |  |  |  |  |
| Kashyap et al. 2014 (15) |  |  |  |  |  |  |  |  |  |  |
| Lönnroos et al. 2012 (16) |  |  |  |  |  |  |  |  |  |  |
| Nishtala et al. 2014 (17) |  |  |  |  |  |  |  |  |  |  |
| Salahudeen et al. 2015 (18) |  |  |  |  |  |  |  |  |  |  |
| Wilson et al. 2010 (19) |  |  |  |  |  |  |  |  |  |  |
| Wilson et al. 2011 (20) |  |  |  |  |  |  |  |  |  |  |
| Wilson et al. 2012 (21) |  |  |  |  |  |  |  |  |  |  |
| * See 'Data extraction' in Methods for an explanation. Notes: a: one star for sample being truly or somewhat representative and one star when non-exposed participants were drawn from same community, b: one star for either assessment through secure record or through structured interview; c: one star if study controlled for most important factor and one star if study controlled for additional factors; d: one star if blindly/independently assessed or obtained through record linkage and in longitudinal studies an additional star if incidence of outcome i.e. absence at baseline was established or verified, e: one star if follow-up was sufficiently long and one additional star if follow-up was complete or when lost-to-follow-up rate was minor and was unlikely to cause bias. | | | | | | | | | | |

**References**

1. Best O, Gnjidic D, Hilmer SN, Naganathan V, McLachlan AJ. Investigating polypharmacy and drug burden index in hospitalised older people. Intern Med J. 2013 Aug;43(8):912-8.

2. Bosboom PR, Alfonso H, Almeida OP, Beer C. Use of Potentially Harmful Medications and Health-Related Quality of Life among People with Dementia Living in Residential Aged Care Facilities. Dement Geriatr Cogn Dis Extra. 2012 Jan;2(1):361-71.

3. Cao YJ, Mager DE, Simonsick EM, Hilmer SN, Ling SM, Windham BG, et al. Physical and cognitive performance and burden of anticholinergics, sedatives, and ACE inhibitors in older women. Clin Pharmacol Ther. 2008 Mar;83(3):422-9.

4. Gnjidic D, Bell JS, Hilmer SN, Lonnroos E, Sulkava R, Hartikainen S. Drug Burden Index associated with function in community-dwelling older people in Finland: a cross-sectional study. Ann Med. 2012a Aug;44(5):458-67.

5. Gnjidic D, Cumming RG, Le Couteur DG, Handelsman DJ, Naganathan V, Abernethy DR, et al. Drug Burden Index and physical function in older Australian men. Br J Clin Pharmacol. 2009 Jul;68(1):97-105.

6. Gnjidic D, Le Couteur DG, Abernethy DR, Hilmer SN. Drug burden index and beers criteria: impact on functional outcomes in older people living in self-care retirement villages. J Clin Pharmacol. 2012b Feb;52(2):258-65.

7. Gnjidic D, Le Couteur DG, Naganathan V, Cumming RG, Creasey H, Waite LM, et al. Effects of drug burden index on cognitive function in older men. J Clin Psychopharmacol. 2012c Apr;32(2):273-7.

8. Hilmer SN, Mager DE, Simonsick EM, Cao Y, Ling SM, Windham BG, et al. A drug burden index to define the functional burden of medications in older people. Arch Intern Med. 2007 Apr 23;167(8):781-7.

9. Lowry E, Woodman RJ, Soiza RL, Hilmer SN, Mangoni AA. Drug burden index, physical function, and adverse outcomes in older hospitalized patients. J Clin Pharmacol. 2012 Oct;52(10):1584-91.

10. Mangoni AA, van Munster BC, Woodman RJ, de Rooij SE. Measures of anticholinergic drug exposure, serum anticholinergic activity, and all-cause postdischarge mortality in older hospitalized patients with hip fractures. Am J Geriatr Psychiatry. 2013 Aug;21(8):785-93.

11. Dauphinot V, Faure R, Omrani S, Goutelle S, Bourguignon L, Krolak-Salmon P, et al. Exposure to anticholinergic and sedative drugs, risk of falls, and mortality: an elderly inpatient, multicenter cohort. J Clin Psychopharmacol. 2014 Oct;34(5):565-70.

12. Gnjidic D, Hilmer SN, Blyth FM, Naganathan V, Cumming RG, Handelsman DJ, et al. High-risk prescribing and incidence of frailty among older community-dwelling men. Clin Pharmacol Ther. 2012d Mar;91(3):521-8.

13. Gnjidic D, Hilmer SN, Hartikainen S, Tolppanen AM, Taipale H, Koponen M, et al. Impact of high risk drug use on hospitalization and mortality in older people with and without Alzheimer's disease: a national population cohort study. PLoS One. 2014 Jan 13;9(1):e83224.

14. Hilmer SN, Mager DE, Simonsick EM, Ling SM, Windham BG, Harris TB, et al. Drug burden index score and functional decline in older people. Am J Med. 2009 Dec;122(12):1142,1149.e1-2.

15. Kashyap M, Belleville S, Mulsant BH, Hilmer SN, Paquette A, Tu le M, et al. Methodological challenges in determining longitudinal associations between anticholinergic drug use and incident cognitive decline. J Am Geriatr Soc. 2014 Feb;62(2):336-41.

16. Lonnroos E, Gnjidic D, Hilmer SN, Bell JS, Kautiainen H, Sulkava R, et al. Drug Burden Index and hospitalization among community-dwelling older people. Drugs Aging. 2012 May 1;29(5):395-404.

17. Nishtala PS, Narayan SW, Wang T, Hilmer SN. Associations of drug burden index with falls, general practitioner visits, and mortality in older people. Pharmacoepidemiol Drug Saf. 2014 Jul;23(7):753-8.

18. Salahudeen MS, Hilmer SN, Nishtala PS. Comparison of anticholinergic risk scales and associations with adverse health outcomes in older people. J Am Geriatr Soc. 2015 Jan;63(1):85-90.

19. Wilson NM, Hilmer SN, March LM, Cameron ID, Lord SR, Seibel MJ, et al. Associations between drug burden index and physical function in older people in residential aged care facilities. Age Ageing. 2010 Jul;39(4):503-7.

20. Wilson NM, Hilmer SN, March LM, Cameron ID, Lord SR, Seibel MJ, et al. Associations between drug burden index and falls in older people in residential aged care. J Am Geriatr Soc. 2011 May;59(5):875-80.

21. Wilson NM, Hilmer SN, March LM, Chen JS, Gnjidic D, Mason RS, et al. Associations between drug burden index and mortality in older people in residential aged care facilities. Drugs Aging. 2012 Feb 1;29(2):157-65.
